# AAV Vectors for FRET-Based Analysis of Protein-Protein Interactions in Photoreceptor Outer Segments

**DOI:** 10.3389/fnins.2016.00356

**Published:** 2016-07-28

**Authors:** Elvir Becirovic, Sybille Böhm, Ong N. P. Nguyen, Lisa M. Riedmayr, Verena Hammelmann, Christian Schön, Elisabeth S. Butz, Christian Wahl-Schott, Martin Biel, Stylianos Michalakis

**Affiliations:** ^1^Department of Pharmacy – Center for Integrated Protein Science Munich (CiPSM), Ludwig-Maximilians-Universität MünchenMunich, Germany; ^2^Department of Pharmacy – Center for Drug Research, Ludwig-Maximilians-Universität MünchenMunich, Germany

**Keywords:** fluorescence resonance energy transfer, FRET, adeno-associated viral vectors, AAV, protein-protein interaction, photoreceptor, outer segment

## Abstract

Fluorescence resonance energy transfer (FRET) is a powerful method for the detection and quantification of stationary and dynamic protein-protein interactions. Technical limitations have hampered systematic *in vivo* FRET experiments to study protein-protein interactions in their native environment. Here, we describe a rapid and robust protocol that combines adeno-associated virus (AAV) vector-mediated *in vivo* delivery of genetically encoded FRET partners with *ex vivo* FRET measurements. The method was established on acutely isolated outer segments of murine rod and cone photoreceptors and relies on the high co-transduction efficiency of retinal photoreceptors by co-delivered AAV vectors. The procedure can be used for the systematic analysis of protein-protein interactions of wild type or mutant outer segment proteins in their native environment. Conclusively, our protocol can help to characterize the physiological and pathophysiological relevance of photoreceptor specific proteins and, in principle, should also be transferable to other cell types.

## Introduction

The outer segments (OS) of rod and cone photoreceptors are highly specialized cilia harboring all proteins involved in light-induced phototransduction (Arshavsky and Burns, 2012). Mutations in genes encoding these proteins lead to untreatable diseases that severely impair rod and/or cone structure and/or functionality (Berger et al., 2010). The majority of these genes encode proteins that are organized in large macromolecular complexes assembled by networks of homomeric and/or heteromeric protein-protein interactions (Roepman and Wolfrum, 2007). To decipher pathomechanisms underlying the retinal disorders and to develop appropriate treatments, the identification and characterization of these networks is inevitable. So far, the vast majority of OS protein-protein interaction studies in photoreceptors were performed using classical biochemical techniques (Goldberg et al., 1995; Loewen and Molday, 2000; Poetsch et al., 2001; Jastrzebska et al., 2004; Michalakis et al., 2011; Knepp et al., 2012). However, the biochemical techniques have several limitations: (i) They strongly rely on the availability of antibodies with high affinity and specificity, (ii) they do not necessarily reflect “true” interactions since, due to the tissue homogenization, some proteins may randomly interact even though they are spatially separated *in vivo*, (iii) they do not allow for dynamic measurements, and (iv) exact quantification of interactions is rather difficult as many different technical parameters which might influence the interaction must be controlled rigorously.

With regard to these obstacles, FRET is superior to the biochemical approaches. One important constraint of FRET is the fact that it requires the co-expression of the fluorescently tagged molecules in a given cell type. This can be easily handled in heterologous expression systems, however, in complex organisms like mammals the application of FRET has been hampered by the laborious and time consuming generation of transgenic animals. Therefore, only a few FRET-based studies were performed *in vivo*, most of them using FRET biosensors for analysis of spatiotemporal dynamics of small signaling molecules (Hovan et al., 2010; Hirata et al., 2012; Wen et al., 2013; Kumagai et al., 2014). Here, we describe a protocol for systematic monitoring of protein-protein interactions without the necessity of generating transgenic animals. The technique utilizes adeno-associated virus (AAV) vectors that are valuable tools for *in vivo* gene transfer into brain and retinal neurons (Schön et al., 2013; Murlidharan et al., 2014; Trapani et al., 2014; Zacchigna et al., 2014). AAVs lead to long-term ectopic expression of the transgene in mammals and thus can be used as a rapid alternative to the generation of transgenic animals. We have already used AAV mediated viral gene expression in retinal neurons in our previous studies (Becirovic et al., 2014, 2016; Nguyen et al., 2016).

**Figure 1:**
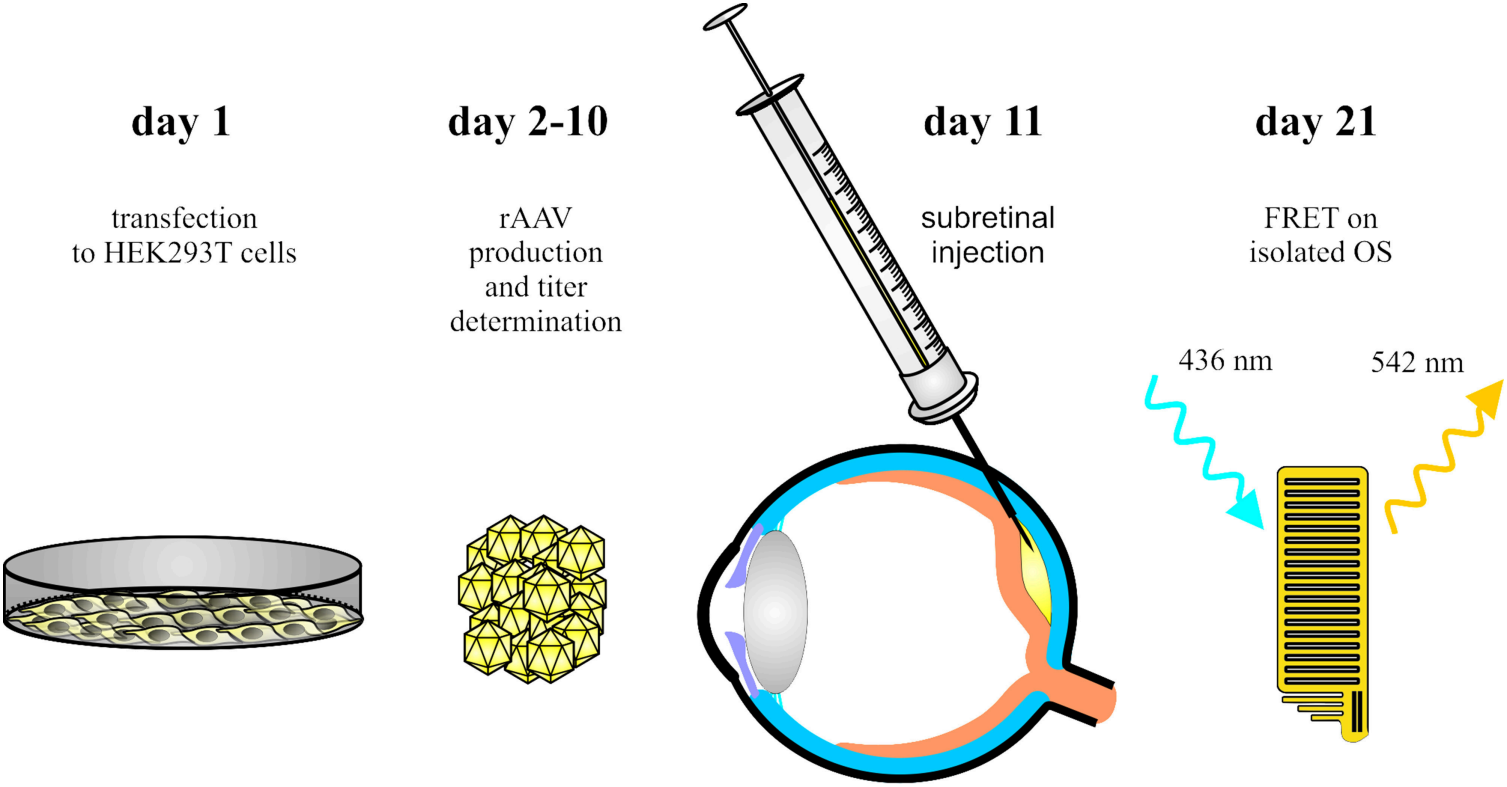
**Schematic overview of the main steps of the procedure**. AAV vectors encoding the FRET fusion proteins are produced in HEK293T cells (day 1–10). Single AAV vectors and combinations of AAVs encoding the FRET partners are delivered into the subretinal space of wildtype mice (day 11). Ten days later, OS are isolated for *ex vivo* FRET measurements.

The protocol allows for rapid and robust measurements of sensitized acceptor emission FRET on isolated virally transduced murine photoreceptor OS. The complete procedure can be performed within 3 weeks on wild type animals and requires only limited expertise and special equipment (Figure 1). We show that this method is suitable for the detection and quantification of protein-protein interactions of proteins involved in the pathogenesis of hereditary degenerative retinal diseases. We propose the application of this method for FRET-based protein-protein interaction studies in other primary cilia of many cell types to study the pathogenesis of other ciliopathies.

### Experimental procedure

The genes of interest encoding each of the protein-protein interaction partners are C- and/or N-terminally fused to the genes encoding citrine or cerulean, two FRET-optimized derivatives of YFP and CFP, respectively (Griesbeck et al., 2001; Rizzo et al., 2004). Each of these constructs is then cloned into the multiple cloning site of a rAAV *cis*-plasmid containing the rod photoreceptor specific human rhodopsin (hRHO) (Allocca et al., 2007) or murine short wavelength opsin (mSWS) promoter (Michalakis et al., 2010a), a Woodchuck hepatitis virus posttranscriptional regulatory element (WPRE), and a bovine growth hormone polyadenylation signal (BGH pA). Subsequently, each rAAV *cis* plasmid is co-transfected with the *trans* (AAV helper and Adeno helper) plasmids into HEK293T cells using triple Ca^2+^ phosphate transfection. rAAV particles are then purified in a multistep procedure that yields high titers of approx. 10^13^ vector genomes (vg) per ml. For FRET measurements, 1 × 10^9^ total vg of each construct is delivered into the subretinal space of anesthetized mice. For co-transduction experiments of two titer-matched constructs a total number of 2 × 10^9^ total vg is injected. The co-transduction efficiency was up to 90% in rods and up to 30% in cones after co-delivery of mixtures of two rAAVs. The isolation of rod or cone OS was performed 10–14 days post-injection using a modified version of the mechanical share method (Mitchell et al., 2009). Three cube FRET measurements were carried out using a slightly modified version of the protocol described previously (Erickson et al., 2003; Shaltiel et al., 2012; Ben Johny et al., 2013). The isolated OS were stable for several hours after isolation, which in principle also allows for dynamic FRET measurements of single interactions.

Using this method, we were able to confirm several homomeric and heteromeric protein-protein interactions in rod OS that had been previously observed using other (biochemical) methods. In addition, we also identified novel protein-protein interactions in OS of rods and cones. Furthermore, we demonstrate that FRET is applicable to quantify the effects of disease associated point mutations on protein-protein interactions in these compartments. Conclusively, we show that our FRET protocol enables robust qualitative and quantitative measurements of protein-protein interactions in small cellular compartments like photoreceptor OS.

### Advantages of the method

Compared to FRET and other *ex vivo* measurements of protein-protein interactions, which are currently widely used, this method has several advantages:

- It can be performed within 3 weeks including the production of specific rAAVs. The isolation of OS can be carried out within a few minutes and yields a high quantity of OS of adequate quality and purity.
- It allows for systematic analysis of protein-protein interactions *ex vivo* in a specialized small compartment with preserved native morphology.
- It is principally transferable to analyses of protein-protein interactions in ciliary compartments of other tissues (e.g., respiratory cilia and olfactory cilia). This is of special importance for deciphering the disease mechanisms of a large group of diseases known as ciliopathies.
- As isolated OS are stable for up to 6 h, the method can also be used for dynamic FRET measurements.
- The protocol was established in wild type mice. Thus, it allows for systematic analysis of disease mechanisms of single disease associated point mutations without the time consuming generation of transgenic animals.
- It can in principle be used to determine the relative binding affinities of single interactions.
- The co-transduction efficiency for two constructs was up to 90% for rod OS and up to 30% for cone OS. Hence, a sufficient number of OS co-expressing both proteins of interest can be isolated from a single animal.
- In principle, it can be adapted for the establishment of FRET measurements on retinas of living animals.
- Due to its easy handling our protocol should also be transferable to other tissues or organisms.

### Limitations of the method

There are a few limitations of the method, which can be overcome by appropriate techniques or conditions:

- With respect to the packaging capacity of rAAVs (5 kb) (Trapani et al., 2014), this method seems less suitable for analysis of protein-protein interactions of large proteins. However, there are several approaches showing that larger transcripts can also be packaged into rAAVs, even though with lower efficiencies (Trapani et al., 2014).
- The endogenous wild type proteins in the OS of transduced photoreceptors interfere with the labeled constructs and lead to overall lower FRET efficiencies than expected from *in vitro* experiments (incomplete labeling) (Becirovic et al., 2014). This limitation should be of relevance only if weak interactions are expected. This issue could be overcome by using appropriate single or double knock-out animals, which lack expression of the endogenous (unlabeled) proteins of interest.
- So far, we found no short or long term toxic effects of the fluorophores on the structural integrity of the retina, which is consistent with previous studies (Bennett et al., 1999; Becirovic et al., 2014). However, we cannot exclude that large amounts of specific cerulean- or citrine-tagged proteins could have toxic effects that influence photoreceptor function and morphology. Therefore, to exclude any toxic effects, we suggest using histological analysis of retinal cross sections prior to the actual FRET measurements for each construct.
- In the case of three cube FRET, in addition to the simultaneous delivery of two FRET partners, injections of single FRET constructs are also necessary. They serve as bleed-through and crosstalk controls for the determination of the FRET efficiencies.
- Overexpression of the tagged proteins by strong promoters might result in an artefactual FRET signal. To exclude this possibility, additional controls might be necessary. In this context, the most eligible control is to use point mutations within the protein-protein interaction site of one (or both) FRET partners. If the protein interaction interface is not known, artefactual FRET signal can be excluded by overexpressing other proteins not expected to interact with the respective FRET partner.
- Bulky fluorescent tags could possibly hinder proper trafficking or localization of the fusion protein (Feilmeier et al., 2000). In addition, they might influence the proper folding and the stability of the protein. In turn, incorrect folding may also affect the fluorophore itself and lead to little or no fluorescence (Waldo et al., 1999). If these problems occur, they can be overcome by varying the position of the fluorescent tag within the protein or by placing a short linker sequence between tag and protein.

## Materials and equipment

### Animals

All procedures were performed on P14-21 C57BL/6J mice with permission from local authorities (District Government of Upper Bavaria). Anesthesia was performed by subcutaneous injection of ketamine (40 mg/kg body weight) and xylazine (20 mg/kg body weight). Euthanasia was performed by cervical dislocation.

### Reagents

- Sodium chloride (NaCl; VWR, cat. no. 27810.364)
- Potassium chloride (KCl; Carl Roth, cat. no. 6781.1)
- Magnesium chloride hexahydrate (MgCl_2_ × 6H_2_O; Carl Roth, cat. no. 2189.1)
- Calcium chloride dihydrate (CaCl_2_ × 2H_2_O; VWR, cat. no. 22317.230)
- Glucose monohydrate (Carl Roth, cat. no. 6887.1)
- HEPES sodium salt (VWR, cat. no. A16516.22)
- Sodium hydroxide, 5 M (NaOH; VWR, 28244.295)
- KAPA SYBR FAST Universal x2 qPCR MasterMix (peqlab, cat. no. 07-KK4600-01)
- Poly-L-lysine, 1 mg/ml (Sigma, cat. no. P2636)
- Polybrene/Hexadimethrine bromide (Sigma, cat. no. 107689)
- Dextran (Sigma, cat. no. 95771)
- BES sodium salt (Sigma, cat. no. B2891)
- AAV *cis* (for cloning of the expression cassette) and *trans* plasmids (encoding for Cap, Rep and adenoviral helper genes) can be obtained from various sources including Penn Vector Core (http://www.med.upenn.edu/gtp/vectorcore) or from UNC Vector Core (http://www.med.unc.edu/genetherapy/vectorcore, see also Ref. Grieger et al., 2006)
- Benzonase (VWR, cat. no. 1.01695.0001)
- OptiPrep (Progen, cat. no. 1114542)
- Disodium hydrogen phosphate (Na_2_HPO_4_; Sigma, cat. no. S9763)
- Sodium dihydrogen phosphate (NaH_2_PO_4_; Sigma, cat. no. S5011)
- Tris(hydroxymethyl)aminomethane (TRIS; VWR, cat. no. 71003-490)
- Phenol red (Sigma, cat. no. P3532)
- Tween 20 (Sigma, cat. no. P2287).

### Equipment for rAAV preparation and subretinal injection

- Vortexer
- Microcentrifuge tubes, 1.5 ml
- Microcentrifuge tubes with screw cap, 1.5 ml
- ibidi μ-dish, 35 mm, low (ibidi, cat. no. 80131)
- Room temperature bench top centrifuge (1.5 ml tube rotor)
- Petri dishes, 3 cm
- Forceps
- Polystyrene tubes, 50 ml
- Cell culture dishes, 15 cm
- Cell scraper (VWR, cat. no. 734-1111)
- Liquid nitrogen
- Quick-Seal polypropylene tubes, 39 ml (Beckman, cat. no. 342141)
- Glass pipettes
- Peristaltic pump MINIPULS 3 (Gilson, cat. no. n/a)
- Quick-Seal Cordless Tube Topper kit, 50 Hz (Beckman, cat. no. 358313)
- Beckman Coulter J2-MC high speed centrifuge (Beckman, cat. no. n/a)
- JA-10 Rotor, Fixed Angle, Aluminum, 6 × 500 ml, 10,000 rpm, 17,700 × g (Beckman, cat. no. 369687)
- Beckman Coulter Optima LE-80K ultracentrifuge (Beckman, cat. no. n/a)
- 70 Ti Rotor, Fixed Angle, Titanium, 8 × 39 mm, 70,000 rpm, 504,000 × g (Beckman, cat. no. 337922)
- Syringe needle, 21-gauge
- Syringe, 5 ml
- Sterile syringe filters with acrylic housing, 0.2 μm, cellulose acetate (VWR, cat. no. 28145-477)
- HiTrap Q FF sepharose column, 5 ml (GE Healthcare, cat. no. 17-5156-01)
- Superloop, 50 ml (GE Healthcare, cat. no. 19-7850-01)
- ÄKTAprime plus chromatography system (GE Healthcare, cat. no. 11-0013-13)
- PrimeView 5.31 software (GE Healthcare, cat. no. 28-9949-61)
- Amicon Ultra-4 centrifugal filter units, 100 kDa (Millipore, cat. no. UFC810024)
- LightCycler 480 multiwell plate 96 (Roche, cat. no. 04729692001)
- LightCycler 480 Sealing Foil (Roche, cat. no. 04729757001)
- LightCycler 480 Instrument II real-time PCR amplification and detection instrument (Roche, cat. no. 05015278001)
- NanoFil syringe, 10 μl (World Precision Instruments, cat. no. NANOFIL)
- NanoFil 34-gauge beveled needle (World Precision Instruments, cat. no. NF34BV-2)
- Nanodrop 2000c UV-Vis spectrophotometer (peqlab, ca. no. 91-ND-2000C)
- Dexpanthenol eye and nose salve, 5% (Bepanthen, Bayer Vital GmbH, cat. no. 1578681)
- OPMI 1 FR pro surgical microscope (Zeiss, cat. no. n/a)
- Gentamicin 5 mg/g and dexamethasone 0.3 mg/g eye salve (Dexamytrex, Dr. Gerhard Mann GmbH, cat. no. 2747789)
- Dissection stereomicroscope Stemi 2000 (Zeiss, cat. no. 495005-0022-000)
- Epifluorescence microscope Axioplan 2 imaging (Zeiss, cat. no. n/a).

### Equipment for three cube FRET measurements

- Inverted fluorescent microscope (Leica, cat. no. DMI6000B)
- HC PL APO 63x/1.40-0.60 oil immersion objective (Leica, cat. no. 11506349)
- Cerulean filter cube, excitation: ET436/20x, Dichroic: T455lp, emission: ET480/40m, transmission rate 93–97% (Chroma Technology, cat. no. 49001)
- Citrine filter cube, excitation: ET500/20x, Dichroic: T515lp, emission: ET535/30m, transmission rate 93–97% (Chroma Technology, cat. no. 49003)
- FRET filter cube, excitation: ET436/20x, Dichroic: T455lp, emission: ET535/30m, transmission rate 93–97% (Chroma Technology, cat. no. 49052)
- DeltaRamX monochromator (Horiba, cat. no. n/a)
- D-104 B microscope photometer (Horiba, cat. no. n/a)
- Xenon short arc lamp type UXL-75XE (Ushio Inc. Japan, cat. no. n/a)
- PMT housing 914 (Horiba, cat. no. n/a)
- R1527 photomultiplier (Horiba, cat. no. n/a)
- FelixGX software (Horiba, cat. no. n/a)
- MATLAB® R2014b (MathWorks. Inc., cat. no. n/a)
- Excel software (Microsoft Corporation, cat. no. n/a).

### Buffers and solutions

**2x BBS transfection solution** Final component concentrations are 45 mM BES, 280 mM NaCl, 1.5 mM Na_2_HPO_4_. Adjust the pH to 6.95 and sterile filtrate. Prepare 50 ml aliquots and store them at 4°C for up to 1 year.

**Lysis Buffer** Final component concentrations are 150 mM NaCl, 50 mM TRIS. Adjust the pH to 8.5 and sterile filtrate. Prepare freshly before use.

**15% iodixanol solution** Final component concentrations are 1x PBS (phosphate buffered saline), 1 mM MgCl_2_, 2.5 mM KCl, 1 M NaCl, 15% Optiprep. Add phenol red (use 1% solution) *ad libitum* until the solution is visibly colored. Store at 4°C for up to 1 week.

**25% iodixanol solution** Final component concentrations are 1x PBS, 1 mM MgCl_2_, 2.5 mM KCl, 25% Optiprep. Add phenol red (use 1% solution) *ad libitum* until the solution is visibly colored. Store at 4°C for up to 1 week.

**40% iodixanol solution** Final component concentrations are 1x PBS, 1 mM MgCl_2_, 2.5 mM KCl, 1 M NaCl, 40% Optiprep. The solution remains uncolored. Store at 4°C for up to 1 week.

**60% iodixanol solution** Final component concentrations are 1 mM MgCl_2_, 2.5 mM KCl, 60% Optiprep. Add phenol red (use 1% solution) *ad libitum* until the solution is visibly colored. Store at 4°C for up to 1 week.

**PBS-MK solution** Final component concentrations are 1x PBS, 1 mM MgCl_2_, 2.5 mM KCl. Sterile filtrate and store at 4°C for up to 1 week.

**Buffer A** Final component concentrations are 20 mM TRIS, 15 mM NaCl. Adjust the pH to 8.5. Store at 4°C for up to 1 week.

**Buffer B** Final component concentration is 2.5 M NaCl. Adjust the pH to 8.5. Store at 4°C for up to 1 week.

**FRET imaging solution** Final component concentrations are 140 mM NaCl, 5 mM KCl,

1 mM MgCl_2_, 2 mM CaCl_2_, 10 mM glucose, 10 mM HEPES sodium salt. Adjust the pH with 5 M NaOH to 7.4. Prepare 50 ml aliquots and store them at −20°C for up to 1 year. Store thawed aliquots at 4°C for up to 1 week.

### FRET imaging setup

**Imaging dishes** We recommend the use of commercially available glass-bottomed cell-culture dishes for imaging. They should be coated with Poly-L-lysine to ensure complete adherence and immobilization of OS. In our hands, the use of pre-coated dishes (e.g., ibiTreat, ibidi) resulted in insufficient OS adherence.

**Inverted epifluorescent microscope, excitation source, and detection** All measurements were performed using a Leica DMI6000B inverted fluorescent microscope equipped with a motorized turret and a 63x oil objective. The microscope contains a motorized filter-wheel enabling rapid switching of filter cubes within 300 ms. Of note, the 40x oil objective is also suitable for FRET measurements on isolated OS. The fluorescent intensity is detected by a photomultiplier detection system including a photomultiplier tube (Horiba). As excitation source, we use a DeltaRam monochromator containing a 150W xenon high stability lamp. The use of a conventional LED lamp is also suitable. However, it is crucial to achieve a stable uniform illumination.

**Excitation filter, emission filter, and dichroic mirror** The following filter cubes are present in our microscope: A donor cube containing a cerulean excitation filter, T455lp dichroic mirror and a cerulean emission filter; an acceptor cube with a citrine excitation filter, T515lp dichroic mirror, and a citrine emission filter. Furthermore a FRET cube containing a cerulean excitation filter, T455lp dichroic mirror and a citrine emission filter. In case of using a monochromator as excitation source, the excitation filters are optional.

**Software and data acquisition** For photometric FRET measurements, we employ the three cube FRET method (Erickson et al., 2003; Shaltiel et al., 2012; Ben Johny et al., 2013) that utilizes the detection of fluorescence intensities from a specimen using three filter cubes. The excitation source (DeltaRam monochromator) and appendant excitation parameters (e.g., wavelength, pulse duration) are thereby controlled via the software FelixGX, whereas the filter switch is done manually. Fluorescence intensity signals are acquired with the FelixGX software and deciphered with a custom-written MATLAB script. Subsequent analysis is performed using Excel or an adequate data processing software.

## Stepwise procedures

### Production and titer determination of rAAVs

#### Transfection, harvesting, and lysis of HEK293T cells

The production and handling of recombinant pseudotyped AAV2-derived vectors is subject to biosafety level 1. For a detailed protocol on AAV production, see ref. (Grieger et al., 2006). 24 h before transfection, confluent 15-cm-dishes of HEK293T cells were split into 15 15-cm-dishes for each construct. For calcium phosphate transfection, the components listed in Table 1 (the amounts are adjusted for transfection of 15 15-cm-dishes) were added in a 50-ml polystyrene tube. The following two equations are used to calculate the amount of pAd Helper plasmid and AAV2/8 Cap/Rep plasmid needed for the pre-transfection mix described in Table 1, respectively:

$$\begin{array}{l} x\;\left(amount\;of\;pAd\;Helper\right)\;=\;\frac{m\;\left(transgene\;AAV\right)\;\times \;MM\;\left(pAd\;Helper\right)}{MM\;\left(transgene\;AAV\right)}\;=\;\frac{270\;\mu g\;\times \;9509\frac{g}{mol}}{MM\;\left(transgene\;AAV\right)}\\ y\;\left(amount\;of\;AAV2∕8\text{ Rep}∕\text{Cap}\right)\;=\;\frac{m\left(transgene\;AAV\right)\;\times \;MM\left(AAV2∕8\text{ Rep}∕\text{Cap}\right)}{MM\left(transgene\;AAV\right)}\;=\;\frac{\;270\;\mu g\;\times \;4523\frac{g}{mol}}{MM\left(transgene\;AAV\right)} \end{array}$$

*MM* = molar mass of double stranded plasmid.

**Table 1:** **Reagents for a pre-transfection mix of one transgene AAV construct**.

**Reagent**	**Amount**
Transgene AAV *cis* plasmid (Michalakis et al., 2010b; Koch et al., 2012)	270 μg
pAd Helper plasmid (pAdDeltaF6 Auricchio et al., 2001)	x μg
AAV2/5 (Hildinger et al., 2001) or AAV2/8 (Gao et al., 2002) Rep/Cap plasmid	y μg
Polybrene (8 mg/ml)	15 μl
Dextran (10 mg/ml)	1500 μl
2.5 M CaCl2	1500 μl
Double-distilled water	ad 15 ml

The listed amounts are for transfection of 15 15-cm-dishes of HEK293T cells.

While vortexing the pre-transfection mix in the polystyrene tube at maximum speed, 15 ml 2x BBS was added dropwise. The mixture was incubated at room temperature for 4–5 min and 2 ml of the transfection mix was added dropwise to each 15-cm-dish of ~80% confluent HEK293T cells. Transfected cells were then incubated at 37°C and 5% CO_2_ atmosphere for 24 h. Subsequently, the medium from transfected cells was replaced and the cells were incubated at 37°C and 10% CO_2_ atmosphere for additional 24 h. 48 h post transfection, the cells were harvested by scraping them from each 15-cm-dish. Cell suspensions were collected from 15 dishes in a 500-ml-centrifuge-tube. The cells were centrifuged at 2000 × g for 15 min at 4°C (4000 rpm in a J2-MC Beckman centrifuge using a JA-10 rotor). After medium removal, the cell pellet was gently resuspended in 7.5 ml lysis buffer (volume for cell pellet harvested from 15 15-cm-dishes) and transferred into 50-ml-polystyrene-tubes. The cell suspension was shock-freezed in liquid nitrogen and subsequently thawed at 37°C. Freeze-thaw cycle was repeated two additional times. The resulting cell suspension can be stored at −80°C overnight.

#### Gradient centrifugation and purification of rAAVs

Benzonase was added to the thawed cell suspension to a final concentration of 50 U/ml followed by an incubation at 37°C for 30 min. Subsequently, the cells were pelleted via centrifugation at 2000 × g for 25 min at 4°C. The virus-containing supernatant was transferred into a Beckman Quick-Seal polypropylene tube. To form the iodixanol gradient, a sterile, long glass pipette, and a Gilson MINIPULS3 pump were used. First, the virus-containing phase was underlaid with 7 ml 15% iodixanol, then with 5 ml 25% iodixanol, followed by 5 ml 40% iodixanol, and at last with 6 ml 60% iodixanol. To avoid mixing of the layers, the pump should be run at the slowest speed. Polypropylene tubes were filled up and accurately balanced with PBS-MK. Prior to centrifugation, the tubes were sealed with the Beckman Tube Topper. Centrifugation was performed at 361.000 × g for 1 h 45 min at 18°C (70.000 rpm in an Optima LE-80K Beckman ultracentrifuge using a 70 Ti rotor). During centrifugation, the virus-containing fraction should accumulate in the 40% phase. After centrifugation, the tube was pierced at the top near the seal for pressurization. To collect the virus-containing phase, a 21-gauge-needle was used with a 5-ml-syringe to pierce the tube through the side at the lower end of the 40–60% interface. During this step, the open site of the needle tip should face the 40% phase. Approximately 5 ml of the 40% phase were collected. The virus-containing phase can be stored in a 15-ml-polystyrene-tube at −80°C until proceeding with the purification step.

For purification, the virus-containing phase was thawed on ice. HiTrap Q FF sepharose column and superloop were connected with the ÄKTAprime plus chromatography system. For collection of the purified fractions, 1.5-ml-centrifuge-tubes were loaded into the tube rack. Dropsync unit was adjusted to position 1. The HiTrap Q FF sepharose column was equilibrated with 25 ml of buffer A at 10 ml/min flow rate. Manual run mode was selected with 1.0 ml/min flow rate, 1 ml fraction size, and the measuring curves were put to starting point via autozero. Virus phase was diluted 1:1 with buffer A prior to application to the ÄKTAprime plus system with a 10 ml syringe to fill the superloop. The virus dilution from the superloop was injected into the ÄKTAprime plus system and 1 ml fractions were collected in 1.5-ml-tubes. During this step, the UV and conductance curves should be monitored via the Äktaprime software. When the conductance curve has reached basal values, the ÄKTAprime plus system was switched to 100% buffer B at 10 ml/min flow rate and 0 ml fraction size to purge the sepharose column from the remaining virus dilution. To wash out the remaining salt from the system, sterile, double-distilled water was used at 10 ml/min flow rate. When the conductance curve reached zero, the washing step was continued for additional 5 min. All of the collected 1-ml fractions within the plateau phase of the conductance curve were pooled. The purified and pooled virus fractions can be stored in a 15-ml-polystyrene-tube at −80°C until continuing with the virus concentration step.

#### Concentration of rAAVs

For virus concentration, the purified virus fraction was first thawed on ice. 4 ml of the fraction was then transferred to an Amicon centrifugal filter unit and was centrifuged at 2000 × g for 10 min at 20°C (4000 rpm in a Beckman centrifuge using a JA-10 rotor). The flow-through was discarded and the cycles of 4 ml virus fraction transfer and subsequent centrifugation were repeated until the complete virus was loaded to the column and ~500 μl remained in the filter unit. The concentrated virus was washed with 1 ml 0.014% Tween/PBS-MK by pipetting up and down five times. The virus suspension was then centrifuged at 2000 × g at 20°C until 100 μl of concentrated virus suspension remained in the filter unit. The supernatant was aliquoted and stored in 1.5-ml screw cap tubes. Virus suspensions were stored at −80°C until determination of virus titer and subretinal injection.

#### Titer determination of rAAVs

For titer determination, the virus suspension was diluted 1:500 with double-distilled water. 5 μl of virus dilution, a standard sample, and double-distilled water were used for quantitative real-time PCR according to Tables 2, 3. For standard preparation, a small fragment within the WPRE (woodchuck hepatitis virus posttranscriptional regulatory element) of the pAAV2.1 vector was amplified via PCR. The corresponding primers are listed in Table 4. The PCR product was purified after gel electrophoresis and DNA concentration was determined with a spectrophotometer (Nanodrop 2000c). The concentration of the standard for 10^10^ copies per 5 μl was calculated according to the following equation:

$$\begin{array}{l} c\left(\frac{pg}{\mu l}\right)=\frac{10^{10}\;\times \;660\;\times \;10^{12}\frac{pg}{mol}\;\times \;fragment\;size}{6.022\;\times \;10^{23}\frac{1}{mol}\;\times \;5\mu l} \end{array}$$

*c* is the concentration of the standard for 10^10^ copies per 5 μl;

660 × 10^12^ pg/mol is the mean molar mass of a base pair (deoxyribosyladenosine with deoxyribosylthymidine or deoxyribosylcytidine with deoxyribosylguanosine);

6.022 × 1023/mol is the Avogadro constant;

The size of the amplified WPRE fragment is given in base pairs.

**Table 2:** **Pipetting scheme for quantitative real-time PCR**.

**Reagent**	**Volume (μl)**
Template plasmid (50–100 ng)/standard/double-distilled water (negative control)	5,0
KAPA SYBR FAST Universal x2 qPCR MasterMix	10,0
WPREqF (10 pmol/μl)	1,0
WPREqR (10 pmol/μl)	1,0
Double-distilled water	ad 20,0

**Table 3:** **Program steps of quantitative real-time PCR for titer determination**.

**Step**	**Phase**	**Temperature (°C)**	**Time**	**Cycle numbers**
1	Initial Denaturation	95	10 min	1
2	Denaturation	95	10 s	40
3	Annealing	60	5 s	
4	Elongation	72	20 s	
5	Final elongation	72	5 min	1

**Table 4:** **Primer sequences for quantitative real-time PCR and standard preparation**.

**Primer for amplification of standard fragment**	**Sequence (5′ to 3′)**
WPREqF	AGTTCCGCCGTGGCAATAGG
WPREqR	CAAGGAGGAGAAAATGAAAGCC

A 10-fold serial dilution was made to obtain standards at different concentrations. 5 μl of a standard were used to determine the virus titer via quantitative real-time PCR. Standards were stored at −20°C until use.

### Subretinal injection of rAAV vectors

The procedure of subretinal rAAV injection was carried out as described by (Mühlfriedel et al., 2013) with slight modifications. The NanoFil 34-gauge beveled needle was sterilized before each experiment. The 10-μl NanoFil syringe was preloaded with the virus suspension under exclusion of air bubbles. The mouse was anesthetized via intraperitoneal injection of xylazine (20 mg/kg) and ketamine (40 mg/kg). Five percent dexpanthenol eye salve was applied to the non-injected eye and the anesthetized mouse was placed on a 37°C heating plate. The eye was dilated with 1% atropine and 0.5% tropicamide solution. The eye fundus was focused with a stereomicroscope until blood vessels became clearly visible. The outer layers of the eye (sclera, choroidea, and retinal pigment epithelium) were then penetrated with the needle in a 60° angle. When the needle was visible beneath the retina, 1 μl (containing approx. 10^9^ rAAV particles) of virus suspension was gradually applied freehand. For the co-delivery of two constructs, a 1:1 mixture of titer matched rAAVs was used. The formation of a clearly visible injection bleb indicated a correct application to the subretinal space. The needle was slowly removed from the eye, which was subsequently treated with gentamicin 5 mg/g and dexamethasone 0.3 mg/g eye salve. The anesthetized mouse was placed under a heat lamp and was monitored until awaking from narcosis. If available, optical coherence tomography (OCT) was conducted immediately after subretinal injection to monitor the injection procedure and the degree of subretinal detachment (Mühlfriedel et al., 2013).

### Dissection of murine retinas and isolation of photoreceptor outer segments

**Figure 2:**
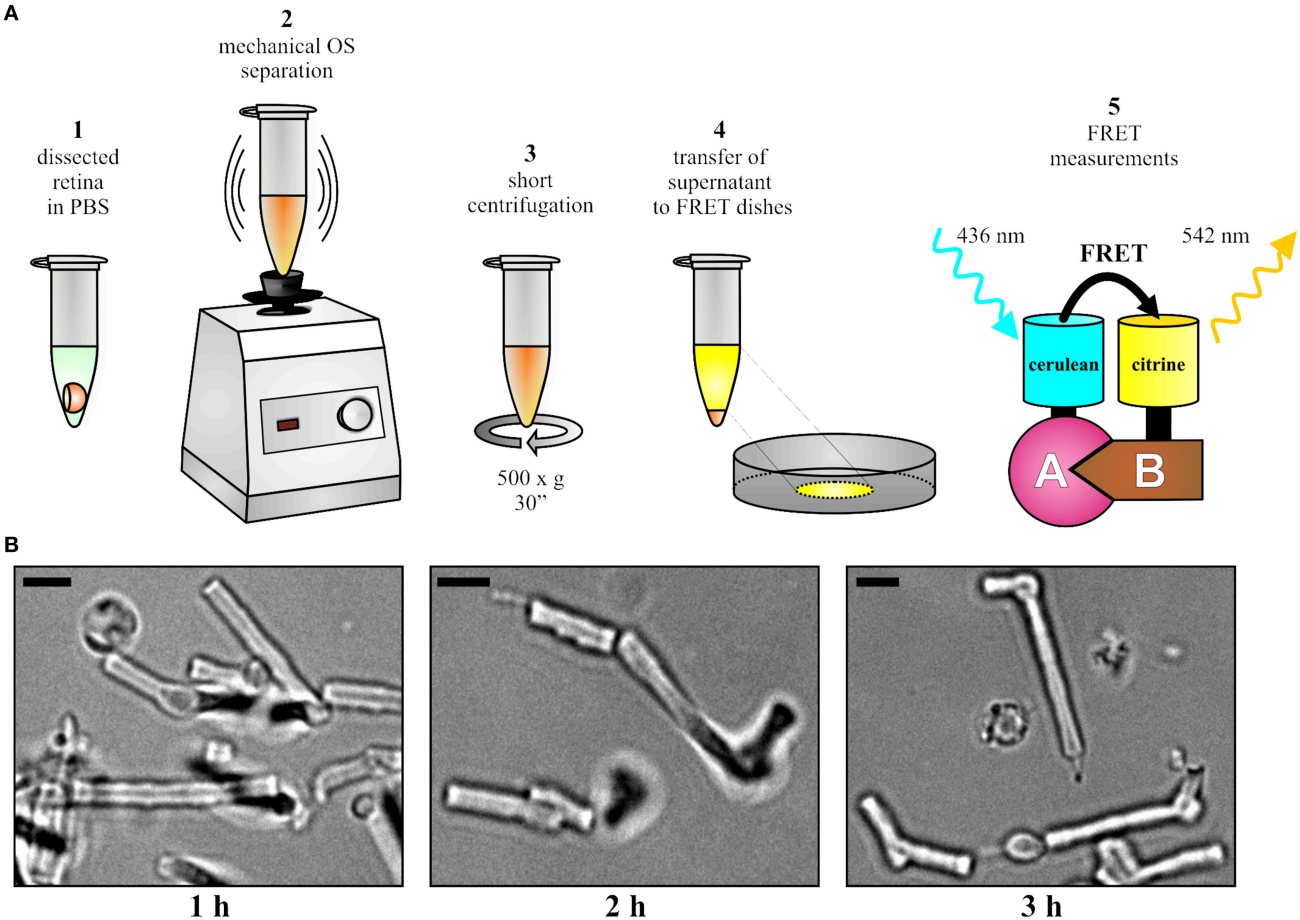
**OS preparation and FRET. (A)** Overview of the protocol for the OS preparation and for the subsequent FRET measurements. **(B)** Representative images of isolated OS at different time points after the isolation. Scale bar, 3 μm.

The mouse was sacrificed 10 days post injection and the injected eye was proptosed by placing blunt forceps around the optic nerve close to its exit from the eye. The globe was then transected along the equator with a sharp razor blade or scalpel and the vitreous body was removed by carefully pushing it out of the incision with a thin needle. The forceps was pushed upwards to detach the retina from the optic nerve and from the pigment epithelium. The upward movement was gradually continued until the retina lay free on the forceps. The isolated retina was transferred into a petri dish filled with phosphate buffered saline (PBS). The expression or co-expression of fluorescent fusion proteins was analyzed by an epifluorescence microscope (Axioplan 2 imaging, Zeiss). As described in Figure 2A, the retina was transferred into a 1.5-ml-microcentrifuge-tube containing 100 μl PBS. In case of weak expression or in case of isolating cone OS, two (or more) retinas can be pooled at this stage to ensure a sufficient number of fluorescent OS. The OS were separated from the retina by vortexing for 15–30 s. Vortexing should not surpass 30 s as excessive shearing disrupts the shape of OS. Afterwards, centrifugation was conducted at 500 × g for 30 s and the supernatant was carefully transferred to a fresh 1.5-ml-microcentrifuge-tube without disturbing the pellet. The supernatant contained the OS fraction suitable for FRET measurements. If desirable, the quality and fluorescence of purified OS can be analyzed (Figure 2B) on a standard epifluorescence or confocal microscope by transferring 5–10 μl of supernatant on microscope slides topped with cover slips.

### FRET measurements on isolated outer segments

#### Setup of FRET microscope, software, and OS sample

Calculation of three cube sensitized acceptor emission FRET requires data for FRET donor (cerulean) only and acceptor (citrine) only samples (Erickson et al., 2003; Shaltiel et al., 2012; Ben Johny et al., 2013). Therefore, in addition to the fluorescence signals from OS co-expressing cerulean and citrine constructs, OS expressing cerulean- or citrine-tagged proteins only must be imaged as well. For optimized signal acquisition, we recommend to start the FRET measurements with OS co-expressing both cerulean- and citrine-tagged FRET partners (hereinafter referred to as FRET sample).

Purified OS solution was diluted in 400 μl FRET imaging solution and was transferred to the center of an imaging dish. FRET measurements should be performed on single OS as fluorescence from adjacent OS might interfere with the measurements. Therefore, the density of OS should be adjusted in order to allow for detection of single fluorescent OS within the defined region of interest on the imaging setup (image-plane pinhole). As the density of transduced OS varies with transduction and purification efficiency, it is necessary to individually adjust the amount of OS for each preparation. The OS were allowed to sediment to the bottom for 10–15 min at room temperature. The fluorescence lamp was turned on at least 30 min prior to the experiment to ensure stable illumination. The image-plane pinhole was set to an appropriate size that allows monitoring of a single OS, which was positioned in the center of the axial beam path. This position must not be changed throughout the entire FRET measurements performed on the same day. The FelixGX software was started to acquire the fluorescence intensities. An oil-immersion and high-resolution objective (40x or higher) was used and immersion medium was applied. The imaging dish was placed onto the sample holder and bright field illumination was used to focus on OS. Individual OS were identified based on their characteristic morphology as shown in Figure 2B.

#### Measurements on the FRET sample

For measurements of FRET samples, OS that express sufficient levels of both citrine and cerulean were used. A fluorescent OS was placed in the center of the optic field and fluorescence from adjacent cells invading the optic field was excluded. Gain and fluorescence intensity settings were adjusted in order to achieve an appropriate signal prior to the first signal acquisition. All subsequent data must be acquired under the same conditions. Fluorescence intensities were collected with the cerulean cube, FRET cube, and citrine cube. The settings for the single cubes are as follows:

For cerulean cube: 426–446 nm excitation filter, T455lp dichroic mirror, and 460–500 nm emission filter; for citrine cube: 490–510 nm excitation filter, T515lp dichroic mirror, and 510–550 nm emission filter; for FRET cube: 426–446 nm excitation filter, T455lp dichroic mirror, and 510–550 nm emission filter.

The data for a single OS was acquired according to the following sequence: cerulean excitation was started and the signals with the cerulean cube followed by the FRET cube were obtained. Then, citrine excitation was carried out and the signal with the citrine cube was obtained. 25–35 individual OS were measured. To subtract the OS autofluorescence, the same three cube protocol as described above was applied to ~10 OS that do not express citrine or cerulean within the corresponding sample.

#### Cerulean-only expressing preparation

OS with varying expression levels of cerulean were used. For each OS, the fluorescence intensity was measured with the cerulean, FRET, and citrine filter cube according to the same protocol as described above for the FRET sample. Data from at least 15 OS were collected. To subtract the OS autofluorescence, the cerulean, FRET, and citrine cube were measured for ~10 OS that do not express cerulean within the same sample.

#### Citrine-only expressing preparation

OS with varying expression levels of citrine were chosen. For each OS, the fluorescence intensity was measured with the cerulean, FRET, and citrine filter cube according to the same protocol as described above for the FRET sample. Data from at least 15 OS were collected. To subtract the OS autofluorescence, the cerulean, FRET, and citrine cube were measured for ~10 OS that do not express citrine within the same sample.

#### Data analysis

The data set consists of three data points per OS, i.e., one intensity value for each filter cube. As controls, we additionally included data points measured from non-fluorescent OS for background subtraction as well as from cerulean-only and citrine-only expressing OS for donor bleed-through and cross-excitation corrections, respectively. The data was transferred to an Excel sheet or an adequate data processing program. For the 10 non-fluorescent OS measured for each of the preparations, i.e. FRET, cerulean-only and citrine-only, the mean intensity values were calculated. These mean values were subtracted from each data point of the particular preparation to adjust for background fluorescence. In order to correct for donor bleed-through, the correction constant *R*_*D*1_ for each of the single data points gathered from the cerulean-only expressing preparation was calculated by dividing the intensities captured with the FRET-cube by those obtained from the cerulean-cube. In order to correct for acceptor cross-excitation, the correction constant *R*_*A*_ for each single data point gathered from the citrine-only expressing preparation was calculated by dividing the intensities captured with the FRET-cube by those obtained from the citrine-cube. The mean value for both constants, *R*_*D*1_ and *R*_*A*_, was calculated. Usually, a value of ~0.25 for cerulean (donor-bleed through) and 0.03 for citrine (acceptor cross-excitation) was obtained in our setup. However, these values might differ with fluorophore variant, protein tag, and imaging setup. The calculated constants were used for donor bleed-through and acceptor cross-excitation to evaluate the FRET ratio (FR) for each single measurement according to the following equation:

$$\begin{array}{l} FR=\frac{S_{FRET}-R_{D1}\cdot S_{cerulean}}{R_{A}\cdot S_{citrine}} \end{array}$$

*S*_*cerulean*_*, S*_*citrine*_, and *S*_*FRET*_ are intensity signals acquired with respective filter cubes (FRET, cerulean, and citrine) in a FRET specimen and *R*_*A*_, *R*_*D*1_ are predetermined factors for calibration issues (*R*_*A*_: Acceptor cross-excitation, *R*_*D*1_: Donor bleed-through). Depending on the imaging setup and, in particular, the filter set used for experiments, a considerable amount of cerulean fluorescence may be detected by the citrine cube. To correct for this, an additional donor bleed-trough correction constant *R*_*D*2_ should be calculated by dividing the cerulean fluorescence intensity obtained with the citrine cube by the one detected with the cerulean cube. Accordingly, the FR equation was adjusted to: (Erickson et al., 2003)

$$\begin{array}{l} FR=\frac{S_{FRET}-R_{D1}\cdot S_{cerulean}}{R_{A}\cdot \left(S_{citrine}-R_{D2}\cdot S_{cerulean}\right)} \end{array}$$

In our FRET setup, *R*_*D*2_ was negligibly small and could thus be ignored. However, it should be rigorously tested for each individual imaging setup. The mean value of the previously obtained data was calculated. The mean value represents the overall *FR*. Note that the *FR* values are instrumentation dependent. FRET efficiencies (*E*_*A*_) (which are independent of the setup used) can be easily calculated from the *FR* using the following equation:

$$\begin{array}{l} E_{A}=\left[FR-1\right]\cdot \frac{\varepsilon_{citrine}\left(436\right)}{\varepsilon_{cerulean}\left(436\right)} \end{array}$$

ε_*citrine*_ and ε_*cerulean*_ are the FRET setup specific average molar extinction coefficients for citrine and cerulean, respectively.

### Troubleshooting

Problems, possible underlying reasons and advices concerning critical steps of the procedure can be found in Table 5.

**Table 5:** **Troubleshooting**.

**Problem**	**Possible Reason**	**Solution**
Low titer.	Transfection reagents do not have sufficient transfection efficiency.	Test transfection reagents for their efficiencies. Requantify plasmid concentration used for transfection.
	pH of HEK293T cell medium is not optimal for transfection.	Remove HEK293T dishes from incubator immediately before transfection.
	The sequence of the ITRs (inverted terminal repeats) within the plasmid is not intact, thus replication and packaging of the rAAV vector is inefficient.	Check for the presence of *SmaI* and/or *Eam1105I* restriction sites within the palindromic ITR sequence. In case of unexpected restriction fragment band pattern, repeat cloning using an intact rAAV vector.
The injection bleb in the subretinal space is not visible under the surgical/stereomicroscope.	The eye shows damages or opacity of the cornea.	Make sure to choose only mice with clear, intact eyes for subretinal injection. Check the eyes under the stereomicroscope.
	Virus suspensions diluted with double-distilled water to adjust the particle concentration do not show optical refraction as clear as the virus suspension in 0.014% Tween/PBS-MK.	Dilute with 0.014% Tween/PBS-MK to adjust virus particle concentration. If the suspension is too viscous, dilute with double-distilled water at a maximum ratio of 1:2 (water-to-virus suspension).
	The injection angle is not optimal.	Adjust the injection angle to ~60°.
Loss of many OS.	Removal of the pigment epithelium and ciliary body after retina isolation with foreceps may result in loss of many OS.	Proceed with the next step without any removal of undesirable tissue.
Protein expression level is too low, thus leading to a too weak or absent fluorescence signal.	Virus suspension is not (completely) injected into the subretinal space.	Make sure to see a clear injection bleb as this indicates the correct injection to the subretinal space. An injection into the upper layers of the eye such as sclera or choroidea causes a bleb outside at the globe. If the needle is intravitreal, it can be clearly and sharply seen under the stereomicroscope. By contrast, a needle in the subretinal space (injection angle at ~60°) appears rather blurred. Keep the injection needle for at least 20 s in the bleb to ensure proper delivery of the desired volume. If possible, perform OCT measurements on the anesthetized mouse after injection to confirm detachment of the retina at the injection position.
	Virus titer has not been determined correctly.	Double check the virus titer. Otherwise, repeat the virus production.

### Time schedule

- Transfection of HEK293T cells: 48 h
- Isolation of rAAV: 2–4 h (varies depending on number of dishes to be harvested)
- Gradient purification and rAAV concentration: 6 h (for 2 different rAAV constructs)
- Titer determination via quantitative real-time PCR: 2 h
- Preparation of standards for titer determination: 2 h
- Subretinal injection: 20 min (per mouse)
- rAAV transduction of murine retina and protein expression: 10 days
- Isolation of photoreceptor OS: 10 min (per eye)
- FRET imaging: 5–9 h (depending on number of retinas to be analyzed)
- Data analysis: 2–3 h (depending on number of measurements to be processed).

## Anticipated results and discussion

**Figure 3:**
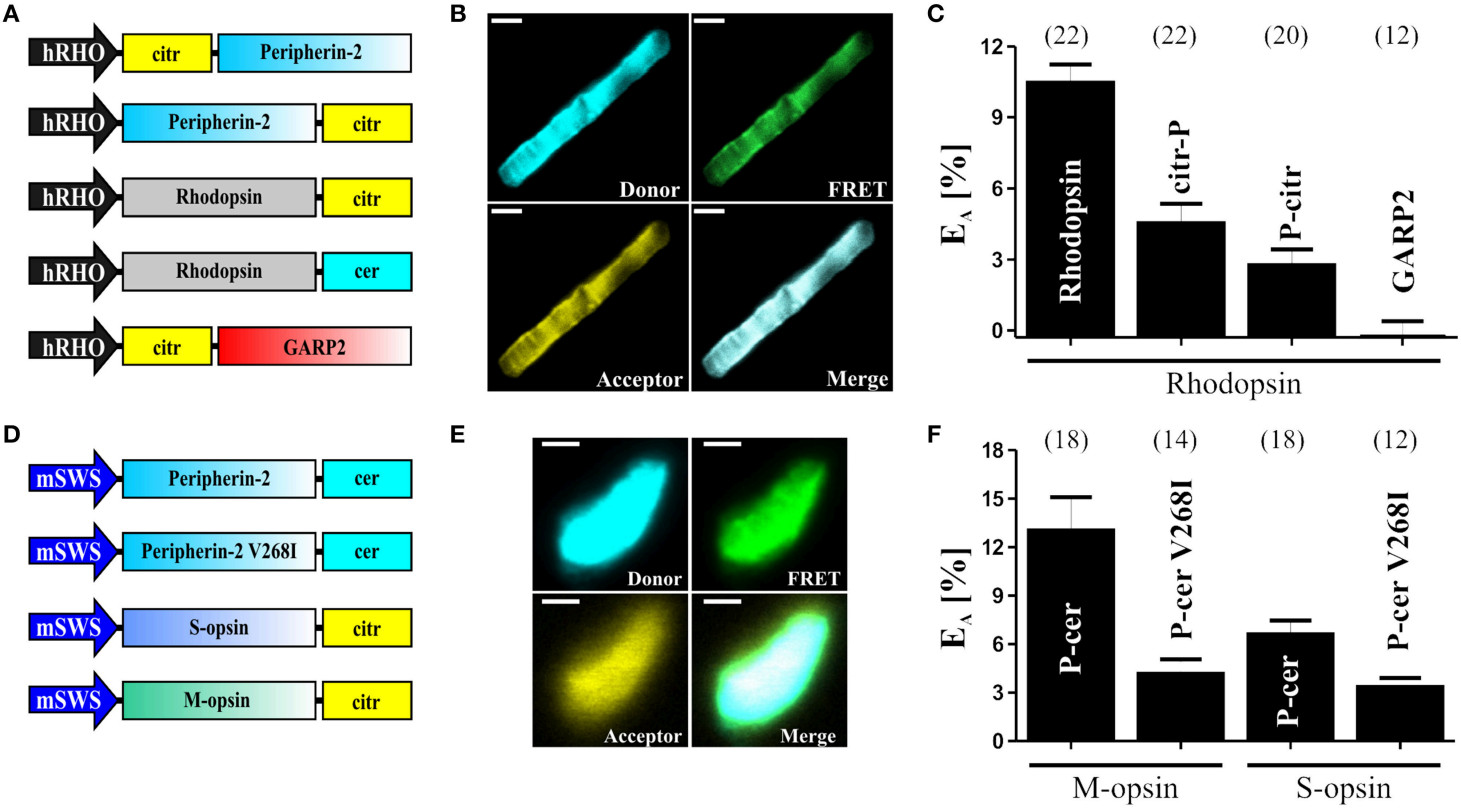
**FRET measurements in isolated photoreceptor OS. (A)** Schematic depiction of the single constructs used for the subretinal injection and for the determination of the FRET efficiencies (*E*_*A*_) shown in **(C)**. **(B)** Representative confocal images of single isolated rod OS co-expressing C-terminally tagged peripherin-2 and rhodopsin. The excitation wavelength and emission filter settings used to obtain the single channels (Donor, FRET and Acceptor) are summarized in the “Stepwise Procedures” section. Scale bar, 1.5 μm. **(C)** Results of the FRET measurements for different FRET pair combinations given as mean values ± standard error of the mean (SEM). Numbers of independent measurements (*n*) are given in brackets. *E*_*A*_ values for the single FRET pairs are as follows: Rho-Rho, *E*_*A*_ = 10.45 ± 0.78; Rho-citr-P, *E*_*A*_ = 4.52 ± 0.82; Rho-P-citr, *E*_*A*_ = 2.76 ± 0.65; Rho-GARP2, *E*_*A*_ = 0.26 ± 0.65; **(D)** Schematic view of the single constructs used for the subretinal injection and for the determination of the FRET efficiencies (*E*_*A*_) shown in **(F). (E)** Representative confocal images of single isolated cone OS co-expressing C-terminally tagged peripherin-2 and cone opsin. Scale bar, 1.5 μm. **(F)** Results of the FRET measurements (given as mean values ± SEM) for different FRET pair combinations as indicated. Numbers of independent measurements (*n*) are given in brackets. *E*_*A*_ values for the single FRET pairs are described elsewhere (Nguyen et al., 2016).

In this protocol, we demonstrate that robust FRET signals can be measured for well-known homomeric and heteromeric interactions of different membrane and soluble proteins in OS of rod and cone photoreceptors. Representative results are found in our previous publications (Becirovic et al., 2014; Nguyen et al., 2016). Selected data from these publications are presented in the modified graphs shown in Figure 3. FRET is given as FRET efficiencies (*E*_A_) which can be easily calculated from the FRET ratios (*FR*) as shown in the “Data Analysis” Section. It is noteworthy to mention that the mean value of *E*_A_ may not represent the maximal FRET efficiency (*E*_A max_). *E*_A max_ can be calculated if a relatively high variability of the cerulean/citrine molar ratios is present in FRET measurements. If this is the case, single FRET values can be plotted against the cerulean/citrine intensity ratios to obtain the binding curves and to calculate *E*_A max_. A comparison of *E*_A max_ between different constructs allows for the determination of relative binding affinities. Since *E*_A max_ is directly proportional to the binding affinity, our method in principle should also allow for comparisons of relative binding affinities of single protein-protein interactions in an isolated subcellular compartment.

Taken together, our protocol enables the measurement of robust FRET signals *ex vivo* in small and highly specialized cellular compartments of mammalian cells like OS of murine photoreceptors. This allows for the systematic analysis of protein-protein interactions in a physiological or pathophysiological context of the photoreceptor biology. In particular, it can be used to analyze the effects of disease-associated mutations on protein-protein interactions. OS of photoreceptors are modified cilia. Ciliopathies encompass a very large group of genetic disorders compromising the functional or structural integrity of cilia. Since the isolation of ciliary compartments from other tissues (i.e. kidney, lung, brain, olfactory epithelium) is well-established (Mitchell et al., 2009), our protocol should also be transferable to analyses of protein-protein interactions in any other ciliated tissue. In non-dividing cells, rAAVs lead to an episomal and long-term expression of the respective gene for up to several years (Trapani et al., 2014). Thus, FRET measurements can be performed on several time points during the development of the tissue. Finally, our protocol could also provide an experimental basis for the establishment of FRET measurements in the retina of living animals by means of fundus ophthalmoscopy combined with e.g., conventional fluorescence imaging, 2-photon imaging, multiphoton FLIM-FRET, or near infrared-FRET fluorescence lifetime imaging (Abe et al., 2013; Johnsson et al., 2014).

## Author contributions

EB designed the protocol with contribution from SM. EB, SB, ON, LR, VH, CS, and ESB performed the experiments, EB, SB, ON, and ESB analyzed the results. EB, ON, ESB, and SM wrote the manuscript with contribution from CW and MB.

## Funding

This work was supported by the Deutsche Forschungsgemeinschaft (DFG, BE 4830/1 1).

### Conflict of interest statement

The authors declare that the research was conducted in the absence of any commercial or financial relationships that could be construed as a potential conflict of interest. Despite hosting the research topic together with one of the authors of this manuscript, the handling Editor state that the process met the standards of a fair and objective review.

## References

[B1] Abe K. Zhao L. Periasamy A. Intes X. Barroso M. (2013). Non-invasive *in vivo* imaging of near infrared-labeled transferrin in breast cancer cells and tumors using fluorescence lifetime FRET. PLoS ONE 8:e80269. 10.1371/journal.pone.0080269 24278268 PMC3836976

[B2] Allocca M. Mussolino C. Garcia-Hoyos M. Sanges D. Iodice C. Petrillo M.. (2007). Novel adeno-associated virus serotypes efficiently transduce murine photoreceptors. J. Virol. 81, 11372–11380. 10.1128/JVI.01327-07 17699581 PMC2045569

[B3] Arshavsky V. Y. Burns M. E. (2012). Photoreceptor signaling: supporting vision across a wide range of light intensities. J. Biol. Chem. 287, 1620–1626. 10.1074/jbc.R111.305243 22074925 PMC3265842

[B4] Auricchio A. Hildinger M. O'Connor E. Gao G. P. Wilson J. M. (2001). Isolation of highly infectious and pure adeno-associated virus type 2 vectors with a single-step gravity-flow column. Hum. Gene Ther. 12, 71–76. 10.1089/104303401450988 11177544

[B5] Becirovic E. Bohm S. Nguyen O. N. Riedmayr L. M. Koch M. A. Schulze E.. (2016). *In vivo* analysis of disease-associated point mutations unveils profound differences in mRNA splicing of peripherin-2 in rod and cone photoreceptors. PLoS Genet. 12:e1005811. 10.1371/journal.pgen.1005811 26796962 PMC4722987

[B6] Becirovic E. Nguyen O. N. Paparizos C. Butz E. S. Stern-Schneider G. Wolfrum U.. (2014). Peripherin-2 couples rhodopsin to the CNG channel in outer segments of rod photoreceptors. Hum. Mol. Genet. 23, 5989–5997. 10.1093/hmg/ddu323 24963162

[B7] Ben Johny M. Yang P. S. Bazzazi H. Yue D. T. (2013). Dynamic switching of calmodulin interactions underlies Ca2+ regulation of CaV1.3 channels. Nat. Commun. 4, 1717. 10.1038/ncomms2727 23591884 PMC3856249

[B8] Bennett J. Maguire A. M. Cideciyan A. V. Schnell M. Glover E. Anand V.. (1999). Stable transgene expression in rod photoreceptors after recombinant adeno-associated virus-mediated gene transfer to monkey retina. Proc. Natl. Acad. Sci. U.S.A. 96, 9920–9925. 10.1073/pnas.96.17.9920 10449795 PMC22311

[B9] Berger W. Kloeckener-Gruissem B. Neidhardt J. (2010). The molecular basis of human retinal and vitreoretinal diseases. Prog. Retin. Eye Res. 29, 335–375. 10.1016/j.preteyeres.2010.03.004 20362068

[B10] Erickson M. G. Liang H. Mori M. X. Yue D. T. (2003). FRET two-hybrid mapping reveals function and location of L-type Ca2+ channel CaM preassociation. Neuron 39, 97–107. 10.1016/S0896-6273(03)00395-7 12848935

[B11] Feilmeier B. J. Iseminger G. Schroeder D. Webber H. Phillips G. J. (2000). Green fluorescent protein functions as a reporter for protein localization in *Escherichia coli*. J. Bacteriol. 182, 4068–4076. 10.1128/JB.182.14.4068-4076.2000 10869087 PMC94594

[B12] Gao G. P. Alvira M. R. Wang L. Calcedo R. Johnston J. Wilson J. M. (2002). Novel adeno-associated viruses from rhesus monkeys as vectors for human gene therapy. Proc. Natl. Acad. Sci. U.S.A. 99, 11854–11859. 10.1073/pnas.182412299 12192090 PMC129358

[B13] Goldberg A. F. Moritz O. L. Molday R. S. (1995). Heterologous expression of photoreceptor peripherin/rds and Rom-1 in COS-1 cells: assembly, interactions, and localization of multisubunit complexes. Biochemistry 34, 14213–14219. 10.1021/bi00043a028 7578020

[B14] Grieger J. C. Choi V. W. Samulski R. J. (2006). Production and characterization of adeno-associated viral vectors. Nat. Protoc. 1, 1412–1428. 10.1038/nprot.2006.207 17406430

[B15] Griesbeck O. Baird G. S. Campbell R. E. Zacharias D. A. Tsien R. Y. (2001). Reducing the environmental sensitivity of yellow fluorescent protein. Mechanism and applications. J. Biol. Chem. 276, 29188–29194. 10.1074/jbc.M102815200 11387331

[B16] Hildinger M. Auricchio A. Gao G. Wang L. Chirmule N. Wilson J. M. (2001). Hybrid vectors based on adeno-associated virus serotypes 2 and 5 for muscle-directed gene transfer. J. Virol. 75, 6199–6203. 10.1128/JVI.75.13.6199-6203.2001 11390622 PMC114336

[B17] Hirata E. Yukinaga H. Kamioka Y. Arakawa Y. Miyamoto S. Okada T. (2012). *In vivo* fluorescence resonance energy transfer imaging reveals differential activation of Rho-family GTPases in glioblastoma cell invasion. J. Cell Sci. 125(Pt 4), 858–868. 10.1242/jcs.089995 22399802

[B18] Hovan S. C. Howell S. Park P. S. (2010). Forster resonance energy transfer as a tool to study photoreceptor biology. J. Biomed. Opt. 15, 067001. 10.1117/1.3505023 21198205 PMC3014226

[B19] Jastrzebska B. Maeda T. Zhu L. Fotiadis D. Filipek S. Engel A.. (2004). Functional characterization of rhodopsin monomers and dimers in detergents. J. Biol. Chem. 279, 54663–54675. 10.1074/jbc.M408691200 15489507 PMC1351296

[B20] Johnsson A. K. Dai Y. Nobis M. Baker M. J. McGhee E. J. Walker S.. (2014). The Rac-FRET mouse reveals tight spatiotemporal control of Rac activity in primary cells and tissues. Cell Rep. 6, 1153–1164. 10.1016/j.celrep.2014.02.024 24630994 PMC3988842

[B21] Knepp A. M. Periole X. Marrink S. J. Sakmar T. P. Huber T. (2012). Rhodopsin forms a dimer with cytoplasmic helix 8 contacts in native membranes. Biochemistry 51, 1819–1821. 10.1021/bi3001598 22352709 PMC3332060

[B22] Koch S. Sothilingam V. Garcia Garrido M. Tanimoto N. Becirovic E. Koch F.. (2012). Gene therapy restores vision and delays degeneration in the CNGB1(−/−) mouse model of retinitis pigmentosa. Hum. Mol. Genet. 21, 4486–4496. 10.1093/hmg/dds290 22802073

[B23] Kumagai Y. Naoki H. Nakasyo E. Kamioka Y. Kiyokawa E. Matsuda M. (2014). Heterogeneity in ERK activity as visualized by *in vivo* FRET imaging of mammary tumor cells developed in MMTV-Neu mice. Oncogene 34, 1051–1057. 10.1038/onc.2014.28 24632612

[B24] Loewen C. J. Molday R. S. (2000). Disulfide-mediated oligomerization of Peripherin/Rds and Rom-1 in photoreceptor disk membranes. Implications for photoreceptor outer segment morphogenesis and degeneration. J. Biol. Chem. 275, 5370–5378. 10.1074/jbc.275.8.5370 10681511

[B25] Michalakis S. Muhlfriedel R. Tanimoto N. Krishnamoorthy V. Koch S. Fischer M. D.. (2010a). Restoration of cone vision in the CNGA3−/− mouse model of congenital complete lack of cone photoreceptor function. Mol. Ther. 18, 2057–2063. 10.1038/mt.2010.149 20628362 PMC2997579

[B26] Michalakis S. Mühlfriedel R. Tanimoto N. Krishnamoorthy V. Koch S. Fischer M. D.. (2010b). Restoration of cone vision in the CNGA3−/− mouse model of congenital complete lack of cone photoreceptor function. Mol. Ther. 18, 2057–2063. 10.1038/mt.2010.149 20628362 PMC2997579

[B27] Michalakis S. Zong X. Becirovic E. Hammelmann V. Wein T. Wanner K. T.. (2011). The glutamic acid-rich protein is a gating inhibitor of cyclic nucleotide-gated channels. J. Neurosci. 31, 133–141. 10.1523/JNEUROSCI.4735-10.2011 21209198 PMC6622767

[B28] Mitchell K. A. Szabo G. Otero Ade S. (2009). Methods for the isolation of sensory and primary cilia–an overview. Methods Cell Biol. 94, 87–101. 10.1016/S0091-679X(08)94004-8 20362086

[B29] Mühlfriedel R. Michalakis S. Garrido M. G. Biel M. Seeliger M. W. (2013). Optimized technique for subretinal injections in mice. Methods Mol. Biol. 935, 343–349. 10.1007/978-1-62703-080-9_24 23150380

[B30] Murlidharan G. Samulski R. J. Asokan A. (2014). Biology of adeno-associated viral vectors in the central nervous system. Front. Mol. Neurosci. 7:76. 10.3389/fnmol.2014.00076 25285067 PMC4168676

[B31] Nguyen O. N. Bohm S. Giessl A. Butz E. S. Wolfrum U. Brandstatter J. H.. (2016). Peripherin-2 differentially interacts with cone opsins in outer segments of cone photoreceptors. Hum. Mol. Genet. 10.1093/hmg/ddw103. [Epub ahead of print]. 27033727

[B32] Poetsch A. Molday L. L. Molday R. S. (2001). The cGMP-gated channel and related glutamic acid-rich proteins interact with peripherin-2 at the rim region of rod photoreceptor disc membranes. J. Biol. Chem. 276, 48009–48016. 10.1074/jbc.M108941200 11641407

[B33] Rizzo M. A. Springer G. H. Granada B. Piston D. W. (2004). An improved cyan fluorescent protein variant useful for FRET. Nat. Biotechnol. 22, 445–449. 10.1038/nbt945 14990965

[B34] Roepman R. Wolfrum U. (2007). Protein networks and complexes in photoreceptor cilia. Subcell. Biochem. 43, 209–235. 10.1007/978-1-4020-5943-8_10 17953396

[B35] Schön C. Biel M. Michalakis S. (2013). Gene replacement therapy for retinal CNG channelopathies. Mol. Genet. Genomics 288, 459–467. 10.1007/s00438-013-0766-4 23861024

[B36] Shaltiel L. Paparizos C. Fenske S. Hassan S. Gruner C. Rotzer K.. (2012). Complex regulation of voltage-dependent activation and inactivation properties of retinal voltage-gated Cav1.4 L-type Ca2+ channels by Ca2+-binding protein 4 (CaBP4). J. Biol. Chem. 287, 36312–36321. 10.1074/jbc.M112.392811 22936811 PMC3476298

[B37] Trapani I. Puppo A. Auricchio A. (2014). Vector platforms for gene therapy of inherited retinopathies. Prog. Retin. Eye Res. 43C, 108–128. 10.1016/j.preteyeres.2014.08.001 25124745 PMC4241499

[B38] Waldo G. S. Standish B. M. Berendzen J. Terwilliger T. C. (1999). Rapid protein-folding assay using green fluorescent protein. Nat. Biotechnol. 17, 691–695. 10.1038/10904 10404163

[B39] Wen L. Thunemann M. Feil S. Hillenbrand M. Vachaviolos A. Ott T. (2013). Analysis of cGMP signalling with transgenic mice expressing FRET-based cGMP sensors. BMC Pharmacol. Toxicol. 14, 1–2. 10.1186/2050-6511-14-s1-p7623289757

[B40] Zacchigna S. Zentilin L. Giacca M. (2014). Adeno-associated virus vectors as therapeutic and investigational tools in the cardiovascular system. Circ. Res. 114, 1827–1846. 10.1161/CIRCRESAHA.114.302331 24855205

